# The contribution of a MOOC to community discussions around death and dying

**DOI:** 10.1186/s12904-018-0287-3

**Published:** 2018-02-20

**Authors:** Jennifer Tieman, Lauren Miller-Lewis, Deb Rawlings, Deborah Parker, Christine Sanderson

**Affiliations:** 1 College of Nursing and Health Sciences, Flinders University, GPO Box 2100, Adelaide, SA 5001 Australia; 2 Faculty of Health, University of Technology Sydney, Level 7, 235 Jones St, Ultimo, NSW 2007 Australia

**Keywords:** Death attitudes, Palliative care, Community education, Online learning, MOOC

## Abstract

**Background:**

Advances in medicine have helped many to live longer lives and to be able to meet health challenges. However death rates are anticipated to increase given the ageing population and chronic disease progression. Being able to talk about death is seen to be important in normalising death as part of life and supporting preparedness for death. Massive Open Online Courses (MOOCs) provide opportunities for the community to engage in collaborative learning. A 5 week MOOC was developed covering four main topics (language and humour, representations of death, medicalisation of dying, and digital dying) aiming:

- To enable participants to openly and supportively discuss and learn about issues around living, death and dying,
- To explore the normally unheard opinions and views of Australians around death and dying, and
- To determine what effect online learning and discussions offered through the MOOC had on participants’ feelings and attitudes towards death and dying.

**Methods:**

Data was captured on engagement rates in the various MOOC activities. Death Attitudes were measured by five items representing the MOOC’s learning objectives and completed at enrolment and conclusion. MOOC Satisfaction was measured with six items at the end of the MOOC. Descriptive statistics were produced for each variable and Chi-Square Tests of Independence assessed the extent of the relationship between categorical variables. Socio-demographic variables were examined as predictors of the outcome variables of MOOC engagement, MOOC satisfaction, and death attitudes. Ethical approval was received from Flinders University Social and Behavioural Research Ethics Committee (Project No. 7247).

**Results:**

One thousand one hundred fifty six people enrolled in the Dying2Learn MOOC with 895 participating in some way. Enrolees were primarily female (92.1%). Age ranged from 16 to 84 (*mean* = 49.5, *SD* = 12.3). MOOC satisfaction scores were high. Responses to the experience of participating in the MOOC were very positive, with mean scores ranging from 4.3 to 4.6 (aligning with agreement and strong agreement to statements on the value of participating). Death Attitudes were positive at commencement but increased significantly following participation.

**Conclusions:**

The Dying2Learn MOOC provided an environment that enabled open and supportive discussion around death and dying and influenced attitudinal change.

## Background

Public health and medical interventions have meant that for many people there has been an opportunity to live longer and for others the ability to live with diseases which may previously have resulted in deaths at an earlier age. However, as the population continues to age and as chronic diseases advance and progress, death rates will rise [1]. This means for individuals and for societies, there is likely to be increasing exposure to death and dying within their families, their communities and their workplaces. Paradoxically, although death is now more likely to be predictable at the individual level and anticipated at the population level, we are not necessarily better prepared for death and dying [2]. Being able to talk about death is important if people are to be able to understand their health outcomes and to make choices about care options. Being able to discuss death openly may also assist individuals in planning as well as preparing family, friends and colleagues as to what will change over time and what may be needed in the future. However, there are indications that individuals and communities are not comfortable in discussing death and dying [3, 4].

Research has shown a disconnect between people’s preferences for place of care and the reality of where they are cared for and die [5]. There are indications that people are not receiving the end of life care they would prefer despite increasing support for advance care planning and advance directives [6, 7]. The impact of population ageing, progressive diseases and end-of-life needs on hospitals and health care systems has also been recognised. This has led to calls to build community engagement with death and dying to encourage active involvement in decision making, community-based caring and acceptance of death as a natural part of the life cycle. However, assumptions about the availability and the capacity of communities to care for their dying in larger numbers remain untested [8]. Work commitments, geographic separation, and other social and personal obligations may reduce caring availability within the community [9]. Patterns of deterioration associated with the end phases of life may also add complexity to the caring regimes already in place for families caring for people with chronic progressive conditions such as heart failure, Chronic Obstructive Pulmonary Disease (COPD) or dementia [10]. However, if as a society we are not able to talk about death and dying in our families and communities and with our care providers, we will not have the confidence, skills and knowledge to engage in our own decision making nor be able to support others in the community [11].

There has been growing recognition of the need to “normalise” death and to provide opportunities for individuals and communities to reconnect with death and dying as a social process rather than a medical outcome [12]. Social initiatives such as death cafes, health promoting palliative care, death education and compassionate communities are all examples of strategies that seek to ensure individuals and systems are aware of, and responsive to, end-of-life issues [13]. Many governments actively support palliative care programs and death awareness initiatives that address the anticipated increases in death and dying [14, 15].

In Australia, the National Palliative Care Strategy [16] looked at four goal areas - Awareness and Understanding, Appropriateness and Effectiveness, Leadership and Governance, and Capacity and Capability. The related “National Palliative Care Projects” funds initiatives and activities to support improvements in the quality of palliative care within Australia in line with the strategy. One of the funded projects is an online Australian palliative care knowledge network, known as CareSearch available at www.caresearch.com.au [17]. CareSearch contributes to palliative care service delivery in a number of ways. First, it builds an understanding of the role of palliative care within the health system and the benefits of palliative care services for those with palliative care needs. Second, it provides evidence based information and resources for health professionals to enhance their clinical practice. Third, it provides patients and carers with quality information and resources to help them in holding discussions with their families and health professionals about their care needs and their preferences. Fourth, it contributes to education and training activities and awareness within the sector. Finally, it encourages research and innovation by providing practical functionalities such as a research data management system and a learning management system that can be used by the palliative care sector to build the evidence base and support continuing professional development [17].

As an online provider, one of the challenges is to develop approaches that can support and engage people virtually. Online learning has been an area of expanding activity within palliative care [18] and is a strategy that had been used successfully by the CareSearch project to support awareness and use of the CareSearch evidence resources. MOOCs, or Massive Open Online Courses, are a relatively new online learning approach. They are freely available short online courses which make use of the digital environment to create socially constructed learning and exchange. They allow for discovery learning, content curation, and peer collaboration and review, providing active, open forums where ideas, issues and subject expertise can be developed, debated, expanded, repurposed and applied [19]. For the CareSearch project, developing a MOOC provided an opportunity to explore the potential of an open access publicly facing technology to build awareness about death and dying. What is learned from the exercise could be used to: inform current practice; suggest new ways of doing business; and suggest different approaches to teaching and learning.

A MOOC, *Dying2Learn*, was scheduled for delivery in 2016. The intended audience was the whole community, not just those involved in palliative care. Accordingly, content planning addressed social cultural considerations around death and dying rather than the specific role of palliative care. The intent of the *Dying2Learn MOOC* was to provide learning resources and a collaborative and supportive environment to facilitate discussions around death and dying. Although there has been some research on community attitudes to death and end of life care [20–22], little is known about Australian attitudes to death and dying, so the MOOC provides a rich environment to understand Australian views and perspectives. The objectives for the MOOC were:

- to enable participants to openly and supportively discuss and learn about issues around living, death and dying,
- to explore the normally unheard opinions and views of Australians around death and dying, and
- to determine what effect online learning and discussions offered through the MOOC had on participants’ feelings and attitudes towards death and dying.

This paper will report on the demographic characteristics of the MOOC participants, their attitudes towards death and dying at the beginning of the course, and any changes to their views about death and dying from the commencement to conclusion of the course. The methodology for this study and an associated research study embedded within the MOOC (reported separately) was approved by Flinders University Social and Behavioral Research Ethics Committee (Project No. 7247). Approval included the use of de-identified content from the MOOC activities for data analysis.

## Methods

### Study overview

The Dying2Learn MOOC was made available to the general public through the OpenLearning platform, with no eligibility restrictions placed on enrolment. Open Learning is an Australian-based company that offers a social online learning platform that delivers over 1500 MOOCs worldwide. The MOOC was promoted widely through CareSearch and related palliative care and consumer networks including death and dying organisations such as the Groundswell Project. To capture consumer attention we used social media platforms, including a Facebook advert, Twitter, and LinkedIn and prepared pieces for consumer-oriented websites and newsletters. The MOOC was also promoted on the OpenLearning website, and on the ‘MOOC List’ website. Interested participants could register to be notified when the MOOC was open for enrolment. The course was created for the Australian public and marketed to this audience, but enrolment was not restricted to those living in Australia. At the point of enrolment, MOOC participants completed a short set of questions regarding their socio-demographic background (gender, age, postcode, education, occupation), and their general attitudes towards death and dying, how it is represented in the media, and how comfortable they feel discussing the topic. After completing enrolment, they were then able to access introductory content prior to the first of 4 weeks of topic based learning resources and activities. The MOOC ended with a week of reflection activities including an evaluation of the MOOC and a repeated assessment of their general attitudes towards death and dying.

### Content development

The *Dying2Learn* MOOC explored social issues around death and dying by looking at how concepts of death and care practices have changed over time, representations of dying and death in the media, and the language used to describe death and dying. The content was developed by a team of academics, educators and researchers with clinical knowledge and expertise in palliative care, associated disciplines and online learning. In the development of course content, the course facilitators were mindful of developing learning outcomes, resources and activities that supported a community-driven approach to death and dying. This meant that the approach to learning was collaborative and exploratory rather than didactic and that the approach to death and dying was through a sociocultural lens rather than a medical or clinical frame. Facilitators helped to guide participants where required, but the participants were viewed as active co-contributors rather than passive recipients of learning. The MOOC content was delivered over 6 weeks, and included an introduction module, 4 core topic modules (released weekly on Monday), and then a final reflections module in the last week. Table 1 summarises the topics and activities covered in each of the 6 weeks of the MOOC. Module 1 focused on how today’s society engages with death and dying through the language we use, humour, public mourning and funerals. Module 2 focused on representations of death and how death is portrayed in history, art, film, TV, and other media. Module 3 focused on the role of medicine in how we die. Module 4 addressed death and its meaning in the digital/internet age. Each module included content such as watching videos, reading articles, and examining media pieces suggested by the MOOC facilitators. Participants engaged in the MOOC’s learning activities by accessing and viewing content, posting comments reflecting on their learning or responding to topic questions, creating submissions (text, links and visual) for the MOOC gallery, and completing private surveys (visible only to that participant and the course facilitators). They could also be involved in discussion boards and live chats, create special interest groups, and share resources.

**Table 1:** MOOC Module Overview and Activity Review

*Activities*	*N Activity Views*	*N Activity Completions*	*% Activity Completions*
*Introduction Module*			
Introduce yourself! [Comment]	1520	680	76.0%
3 Words to describe feelings about death [Submission]	2737	653	73.0%
How did you find us? [Comment]	723	661	73.9%
MOOC experience and motivations [Private Survey]	724	640	71.5%
*Module 1: How we engage with death and dying*			
Reflection on ‘No Laughing Matter’ [Comment]	1221	537	60.0%
Find a Joke for ‘No Laughing Matter’ [Submission]	1912	425	47.5%
Words are not enough - Euphemisms [Submission]	2021	505	56.4%
Reflection on ‘How do people engage with death and dying?’ [Comment]	901	440	49.2%
Design your own roadside memorial/eulogy/headstone [Submission]	1710	395	44.1%
*Module 2: Representations of death*			
Historical death bed paintings [Comment]	1067	396	44.2%
Two films depicting death [Submission]	1549	357	39.9%
Death in TV Dramas and Documentaries [Comment]	636	344	38.4%
Death and dying in other mediums [Comment]	684	337	37.7%
*Module 3: If death is the Problem… is medicine the answer?*			
The best way to go [Submission]	1318	308	34.4%
Reflections on “Being Mortal” [Comment]	816	296	33.1%
Prolonging Life Prolonging Death Live Chat [Comment]	2022	306	34.2%
*Module 4: Digital Dying*			
My Digital Selection [Comment]	743	286	32.0%
Create a Deathwise Communication [Submission]	2227	320	35.8%
*Final Reflections Module*			
3 words describing death Revisited [Submission]	815	227	25.4%
Before I die I want to…. [Comment]	351	228	25.5%
A final Challenge: If I was health minister [Submission]	804	217	24.2%
Reflection Questions [Private Survey]	485	155	17.3%
Spreading the word? [Comment]	328	170	19.0%
Light-bulb moments [Comment]	297	172	19.2%

*Note.* The square brackets [] indicate the way in which participants were able to respond to the module, i.e. via comment, submission or private survey

### Data collection and participants

Information on the participants and on their attitudes to death was collected as part of enrolment process for the MOOC. The participants also completed an evaluation survey and completed the death attitudes questions again as part of reflection activities in the last week of the MOOC. The OpenLearning platform enabled passive collection of MOOC activity. The data were collected by OpenLearning and extracted by them for analysis at the conclusion of the MOOC. Data was fully de-identified prior to analysis.

A total of 1156 people enrolled in the Dying2Learn MOOC with 895 (77.4%) subsequently participating in some content or activity in the MOOC. A total of 210 MOOC participants completed the MOOC evaluation activity in the final week, representing 18.2% of those who initially enrolled in the MOOC, and 23.5% of those who had commenced participation in the MOOC. Of these, 208 had matched data from both the enrolment and the final activity on death attitudes which allowed a pre-post assessment of change in death attitudes.

### Measures

*Socio-Demographic Background* was collected through five questions at the point of MOOC enrolment. Participants were asked to provide their age in years, and the gender they identify with (with 5 options of male, female, Trans, other, or prefer not to disclose). Given the very small number of participants utilizing the latter 3 options (*n* = 14), where gender was included in analyses it was dichotomized to compare males and females (with the remainder coded as missing). Regarding occupation, participants identified themselves on the dichotomous categorization as either a health professional or not a health professional. They were also asked to report their highest level of education they had completed based on a four-category ordinal scale adapted from the Australian Bureau of Statistics 2016 census [23] (some high school, completed high school, trade school/equivalent, and university studies). For the purpose of analysis, education was also dichotomized to compare those with university qualifications to those without university qualifications. Participants’ 4-digit Australian residential postcode was recorded if they lived in Australia, or alternatively the name of the country they resided in if they were not located in Australia. For participants residing in Australia (*n* = 1078), the Socio-Economic Index for Areas (SEIFA) Index of Relative Socio-Economic Disadvantage (IRSD) corresponding to their residential postcode was assigned, which is based on 2011 census data from the Australian Bureau of Statistics [24]. The 2011 Census SEIFA Disadvantage Index ranks 2481 postal areas in Australia according to relative disadvantage. It summarizes a range of information about the economic and social conditions of people and households within each area. Scores based on the 2011 Census can range from 506.3 to 1155.5, with low scores indicating greater disadvantage in that area (i.e., many households with unemployment, low income, no qualifications, and low skilled occupations). Higher scores on the SEIFA Disadvantage index indicate that the postal area has a relative lack of disadvantage (i.e., few households with unemployment, low incomes, no qualifications, and low skilled occupations) [24]. In the present study, SEIFA Disadvantage scores ranged from 744 to 1155.5.

*Death Attitudes* were measured with five items designed for the purpose of evaluating the impact of participation in the Dying2Learn MOOC, and were representative of the learning objectives of the MOOC. These items were presented both at enrolment (baseline) and at the end of the MOOC in a final activity (follow-up), to allow the assessment of change over time in death attitudes. Participants were asked to rate their level of agreement with five statements on a five-point Likert scale of ‘strongly disagree’, ‘disagree’, ‘not sure’, ‘agree’ and ‘strongly agree’. The statements (listed in Table 2) investigated attitudes towards death as a normal part of life, level of comfort in talking about death, and about how death and dying are presented in the mainstream media and social media.

**Table 2:** Socio-demographic Characteristics^a^ and Attitudes to Death^b^

	*All Enrolees with Response Data*		*Enrolled and Commenced (n = 895)*	*Enrolled did not Commence (n = 261)*
	*n*	*M (SD) or %, range*	*M (SD) or %, range*	*M (SD) or %, range*
*Socio-Demographic Characteristics at Enrolment*				
Gender (female)	1156	92.1%	93.1%	91.2%
Age	1148	49.5 (12.3), 16–84	50.1* (12.0), 19–84	47.2* (13.2), 16–79
Located in Australia	1156	93.8%	94.1%	92.7%
SEIFA Disadvantage Index for Australian Postcode	1078	1007.5 (63.2), 744–1155.5	1005.5* (62.1), 808.7–1128.2	1014.6* (66.4), 744–1155.5
Self-identifies as a Health Professional	1154	68.0%	68.3%	67.0%
Has a University Qualification	1154	70.6%	70.8%	70.1%
Highest Level of Education:	1154	–	–	–
Some High School	–	4.6%	4.8%	3.8%
Completed High School	–	8.1%	8.3%	7.7%
Trade school or Equivalent	–	16.6%	16.1%	18.4%
University Studies	–	70.6%	70.8%	70.1%
*Attitudes Towards Death, (possible range 1–5)*				
*Death is a normal part of life*				
Pre-MOOC (Enrolment)	1154	4.6 (0.9), 1–5	4.6 (0.9), 1–5	4.5 (1.0), 1–5
Post-MOOC	210	4.9 (0.4), 2–5	4.9 (0.4), 2-5^c^	–
*I am comfortable talking about death/dying*				
Pre-MOOC (Enrolment)	1154	4.3 (0.9), 1–5	4.3 (0.9), 1–5	4.3 (0.9), 1–5
Post-MOOC	210	4.5 (0.6), 2–5	4.5 (0.6), 2-5^c^	–
*Most people do NOT feel comfortable talking about death/dying*				
Pre-MOOC (Enrolment)	1154	4.0 (0.7), 1–5	4.0 (0.7), 1–5	3.9 (0.7), 1–5
Post-MOOC	210	4.0 (0.8), 1–5	4.0 (0.8), 1-5^c^	–
*Death/dying is presented as a normal part of life in the mainstream media*				
Pre-MOOC (Enrolment)	1154	2.4 (0.9), 1–5	2.4 (0.9), 1–5	2.4 (0.9), 1–5
Post-MOOC	210	2.5 (1.0), 1–5	2.4 (1.0), 1-5^c^	–
*Social media provides different perspectives to mainstream media on death/dying*				
Pre-MOOC (Enrolment)	928^d^	3.4 (0.7), 1–5	3.4 (0.7), 1–5	3.3 (0.7), 1–5
Post-MOOC	210	3.7 (0.8), 1–5	3.7 (0.8), 1-5^c^	–

^a^Socio-demographics for all Enrolled, Enrolled and commenced, and Enrolled not commenced

^b^Attitudes to Death (Pre only for all enrolled and enrolled did not commence and Pre-post for enrolled and commenced)

^c^Based on *n* = 208 with valid Post-MOOC death attitudes data

^d^Due to a temporary technical problem on the MOOC platform, 228 enrolees were not asked this question

*There was a statistically significant difference (*p* <. 05) between enrolees who commenced the MOOC and enrolees who did not. Those who commenced the MOOC were significantly older, and lived in significantly lower SES (SEIFA) areas based on independent samples t-test results. There were no other statistically significant differences between those who commenced the MOOC and those who didn’t on any of the other variables assessed at the point of enrolment

*MOOC Satisfaction* was measured with six items at the end of the MOOC in a final evaluation activity. The items were adapted from evaluations of courses/workshops related to death and palliative care [25, 26]. Participants were asked to respond to six statements on a five-point Likert scale from ‘strongly disagree’ to ‘strongly agree’. This assessed opinions on whether: the MOOC was enjoyable; it met expectations; they would recommend it to others; they would feel comfortable talking about MOOC content to others; and the MOOC had given them a deeper understanding of death, and helped them gain insight into personal beliefs.

*MOOC Engagement* metrics were automatically generated by the OpenLearning MOOC platform, and were extracted from the platform after the closure of the MOOC. The total percentage of course progress gave an indicator of the level of engagement for each participant based on the overall proportion of all content pages accessed and activities completed. A count score on the total number of comments made throughout the MOOC was also provided.

### Statistical approach

This study analyses data based on enrolment in the MOOC, MOOC engagement, and evaluation of the MOOC embedded in a final activity of the course. On completion of the MOOC, data extraction was facilitated by the team at OpenLearning, and was de-identified prior to data analyses. All analyses included a large sample size (ranging from a minimum of *n* = 179 up to *n* = 1156), therefore providing adequate statistical power to the analyses. Descriptive statistics were produced for each variable (means and standard deviations for continuous and ordinal variables, or proportions for categorical variables). Chi-Square Tests of Independence were conducted to assess the extent of the relationship between categorical variables. Socio-demographic variables were examined as predictors of the outcome variables of MOOC engagement, MOOC satisfaction, and death attitudes. The ordinal nature of the MOOC satisfaction and death attitude variables, and the skewed nature of their distributions in this sample meant that non-parametric statistics were the most appropriate method for data analysis. Mann-Whitney U Tests were used to identify statistically significant between-group differences on these ordinal variables where the grouping variable had only two categories (e.g., health professional status). Spearman’s Rank-Order Correlation was used to analyse their associations with continuous socio-demographic scores (age and SEIFA). The Wilcoxon-Signed rank test was used to analyse repeated-measures change over time on the ordinal death attitude variables. For completeness, data analysis was also conducted using the parametric alternatives to these tests, and the resulting conclusions were the same. Data analyses were conducted in IBM SPSS version 23.

## Results

Table 2 outlines the socio-demographic characteristics of those enrolled in the MOOC. The majority of enrolees were female (92.1%). The age of participants ranged from 16 to 84, with a mean of 49.5 (*SD* = 12.3) and 78.2% percent aged 40 years and over, and 20.6% percent aged 60 and over. The majority of participants (68%) self-identified as health professionals, and 70.6% held a university qualification. Less than 5% had not completed high school. Overall, 94% of the enrolees resided in Australia, with the remainder spread across 15 countries, but predominantly from the English-speaking countries of United Kingdom (1.7%), United States (1.4%), New Zealand (1.2%) and Canada (0.8%).

While 1156 people enrolled in the MOOC, 261 of these never accessed any course content. These details of all enrolees, enrolees who commenced the course and enrolees who did not commence are compared in Table 2. On all the variables assessed at the point of enrolment, there was only a statistically significant difference (*p* < .05 as measured by independent samples t-test) between those who commenced the MOOC and those who didn’t, for two variables - age and the SEIFA Index of disadvantaged areas. Those who commenced the MOOC were significantly older, and lived in significantly lower SES (SEIFA) areas compared to those who did not commence the MOOC. There were no significant differences on gender, Australian location, health professional or education status, or on responses to the death attitude questions asked at enrolment.

The MOOC was evaluated in respect to level of engagement by participants as well as pre- and post-MOOC measures of attitudes towards death and dying. Descriptive statistics on attitudes to death are provided in Table 2. It can be seen that attitudes towards (a) death as a normal part of life, (b) feeling comfortable talking about death, and (c) belief that most people do not feel comfortable talking about death, tended to be at the higher end of the agreement scale, with mean scores of at least 4 out of a possible 5 (with 5 indicating strong agreement). Responses to the questions about how death is portrayed in the mainstream media and social media tended more towards the middle of the scale, indicative of being unsure.

The online learning platform provided metrics related to the level of MOOC engagement by participants, including the percentage of course progress and the number of comments made. Overall the course modules pages were viewed 18,216 times, 9872 comments were made in the MOOC and 10,232 tasks were completed. Table 3 provides details on engagement with the MOOC. For enrolled participants who commenced the MOOC, the mean percentage of course progress reached by the conclusion of the MOOC was *M* = 37.4% (*SD* = 31.0). A total of 16% of commencing students completed at least 80% of the MOOC, and 3% (*n* = 27) of the commencing students completed every single aspect of the MOOC (meaning they opened every content page, made comments on all pages and completed all 24 activities). On average, students who commenced the MOOC made 10.6 comments (*SD* = 14.3), with a range of zero to 265. A total of 16.3% of students who commenced the course made 20 or more comments during the course of the MOOC, with *n* = 14 students making in excess of 50 comments, indicating a very high level of engagement in the topic.

**Table 3:** MOOC engagement and post MOOC evaluation for participants who commenced the MOOC

	*M (SD), range*
*MOOC engagement measures (n = 895)*	
MOOC percentage of course progress	37.4 (31.0), 1–100
Number of comments made in MOOC	10.6 (17.2), 0–265
*Post-MOOC Evaluation (possible range 1–5) (n = 206)*	
MOOC was enjoyable	4.6 (0.6), 2–5
MOOC met expectations	4.4 (0.8), 1–5
Would recommend MOOC to others	4.5 (0.6), 2–5
MOOC gave deeper understanding of death	4.4 (0.8), 2–5
Gained personal insight into own beliefs	4.3 (0.8), 1–5
Feel comfortable talking to people about MOOC content	4.6 (0.5), 2–5

At the conclusion of the MOOC in the final reflections week, participants were asked to complete an evaluation of their MOOC experience by answering six survey items. A total of 206 MOOC commencers completed this activity, with the results shown in Table 3. It can be seen that the response to the experience of participating in the MOOC was very positive, with mean scores ranging from 4.3 to 4.6, aligning with agreement and strong agreement to statements on the value of participating in the MOOC. For example, 96.5% of respondents agreed/strongly agreed that the MOOC was enjoyable; 96.1% agreed/strongly agreed that the MOOC met their expectations; 94.6% agreed/strongly agreed that they would recommend the MOOC to others; and 91.2% agreed/strongly agreed that the MOOC gave them a deeper understanding of death. These responses indicate a very high level of satisfaction with the *Dying2Learn* MOOC experience overall.

An examination of any differences in levels of satisfaction with the MOOC based on participants’ demographic characteristics was conducted. For gender, health professional status, and university qualification status, the Mann-Whitney U Tests found no statistically significant differences (*p* > .05), with the exception of males (*M* = 5.0) being slightly more comfortable than females (*M* = 4.54) to talk to other people about the content of the MOOC, *U* = 540, *Z* = − 2.72, *p* = .007). There were no statistically significant associations between age or SEIFA disadvantage and the MOOC satisfaction responses (all Spearman’s Rank Order correlations were less than.10, *p* > .05).

Further analyses were conducted to determine if those enrolees who were more uncomfortable talking about death were less likely to commence the MOOC after enrolment. This was not the case, with those feeling uncomfortable just as likely to commence the MOOC as those who felt comfortable (79% of those who strongly disagreed to feeling comfortable commenced the MOOC versus 77% of those who strongly agreed to feeling comfortable). A Chi-Square Test of Independence indicated that there was no significant association between a person’s level of comfort in talking about death and their MOOC commencement status *χ*^*2*^ (*df* = 4, *n* = 1154) = 3.55, *p* = .47, *phi* = .055. There was also no significant association between a person’s level of comfort in talking about death and whether they formally withdrew from the MOOC, *χ*^*2*^ (*df* = 4, *n* = 1154) = 7.05, *p* = .13, *phi* = .078. None of the enrolees who reported feeling uncomfortable talking about death ended up withdrawing from the course.

Based on the death attitudes questions at enrolment, we also examined the relationship between people’s feelings regarding talking about death and how this related to what they think about how others feel when talking about death. These figures are included in Table 4. A Chi-Square Test of Independence indicated that there was a significant association between a person’s own level of comfort in talking about death and their perception of the level of comfort others felt in talking about death *χ*^*2*^ (*df* = 16, *n* = 1154) = 151.2, *p* = .000. The ordinal-by-ordinal Spearman’s Rank Order correlation was .08, *p* = .009, indicating a small effect. Participants who agree/strongly agree that they are comfortable talking about death are more likely to report they agree/strongly agree that most people do NOT feel comfortable talking about death (e.g. of those who strongly agreed they are comfortable talking about death, 89.7% agreed or strongly agreed that most people do NOT feel comfortable talking about death).

**Table 4:** Comparing views on personal comfort and comfort of others in talking about death and dying (*n* = 1154)

	Most people are NOT comfortable talking about death and dying						
		Strongly Disagree	Disagree	Not Sure	Agree	Strongly Agree	Totals
		*n, %*	*n, %*	*n, %*	*n, %*	*n, %*	*n, %*
I am comfortable talking about death and dying	Strongly Disagree	9 (0.8%)	0 (0.0%)	7 (0.6%)	25 (2.2%)	2 (0.2%)	43 (3.7%)
	Disagree	0 (0.0%)	0 (0.0%)	1 (0.1%)	14 (1.2%)	6 (0.5%)	21 (1.8%)
	Not Sure	0 (0.0%)	1 (0.1%)	7 (0.6%)	58 (5.0%)	10 (0.9%)	76 (6.6%)
	Agree	3 (0.3%)	13 (1.1%)	51 (4.4%)	340 (29.5%)	66 (5.7%)	473 (41.0%)
	Strongly Agree	4 (0.3%)	24 (2.1%)	28 (2.4%)	396 (34.3%)	89 (7.7%)	541 (46.9%)
	*n* and % of Total	16 (1.4%)	38 (3.3%)	94 (8.1%)	833 (72.2%)	173 (15.0%)	1154 (100%)

Table 5 compares the responses to the five death attitude questions at enrolment to the responses given at the end of the MOOC by 208 participants who completed the enrolment questions and the evaluation survey as a final MOOC activity. An examination of Table 5 highlights the changes that occurred over time in participant’s attitudes towards death after they took part in the Dying2Learn MOOC. The first section displays results for the attitude ‘death is a normal part of life’, and indicate a clear skew in attitudes, with 69.7% of participants reporting they strongly agree with this statement at both the enrolment and the conclusion of the MOOC. Furthermore, by the end of the MOOC all but one person (99.5%) agreed or strongly agreed that death is a normal part of life. All of the participants who initially strongly disagreed that death is a normal part of life over the course of the MOOC changed their opinion to strongly agree. Overall, the majority of responses stayed the same (*n* = 159), but more than twice as many positive changes occurred over time than negative changes. A Wilcoxon Signed Rank Test revealed a statistically significant increase in agreement with the statement that ‘death is a normal part of life’ following participation in the *Dying2Learn* MOOC, *Z* = 3.36, *p* < .001, with a small effect size (*r* = .165). The mean score on the ‘death is a normal part of life’ question increased from pre-MOOC (*M* = 4.61) to post-MOOC (*M* = 4.85), and the median remained 5.0 (‘strongly agree’) both pre- and post-MOOC. This clearly indicates that the people who chose to participate in the *Dying2Learn* MOOC overall had very positive attitudes to death as a normal part of life at the beginning of the MOOC. This self-selection bias created a ceiling effect in regards to room for movement in attitudes over the time period involved in the MOOC. Despite this, for those MOOC participants who had lower scores on ‘death is a normal part of life’ at the beginning, they did show a significant increase in positive attitudes at the conclusion of the MOOC.

**Table 5:** Pre-Post MOOC Attitudes Toward Death (*n* = 208)^a^

	Post-MOOC					
Pre-MOOC	Strongly Disagree	Disagree	Not Sure	Agree	Strongly Agree	Total
	n,%	n,%	n, %	n, %	n, %	N, %
*1. Death is a normal part of life*						
Strongly Disagree	0 (0%)	0 (0%)	0 (0%)	0 (0%)	10 (4.8%)	10 (4.8%)
Disagree	0 (0%)	0 (0%)	0 (0%)	0 (0%)	1 (0.5%)	1 (0.5%)
Not Sure	0 (0%)	0 (0%)	0 (0%)	0 (0%)	2 (1%)	2 (1%)
Agree	0 (0%)	0 (0%)	0 (0%)	14 (6.7%)	21 (10.1%)	35 (16.8%)
Strongly Agree	0 (0%)	1 (0.5%)	0 (0%)	14 (6.7%)	145 (69.7%)	160 (76.9%)
n and % of Total	0 (0%)	1 (0.5%)	0 (0%)	28 (13.5%)	179 (86.1%)	208 (100%)
Positive Differences						34
Negative Differences						15
Ties						159
Wilcoxon-Signed Rank Test Z Statistic ^b c^						3.36***
*2. I am comfortable talking about death/dying*						
Strongly Disagree	0 (0%)	0 (0%)	1 (0.5%)	2 (1%)	7 (3.4%)	10 (4.8%)
Disagree	0 (0%)	1 (0.5%)	0 (0%)	2 (1%)	1 (0.5%)	4 (1.9%)
Not Sure	0 (0%)	0 (0%)	2 (1%)	8 (3.8%)	1 (0.5%)	11 (5.3%)
Agree	0 (0%)	2 (1%)	2 (1%)	43 (20.7%)	34 (16.3%)	81 (38.9%)
Strongly Agree	0 (0%)	0 (0%)	1 (0.5%)	21 (10.1%)	80 (38.5%)	102 (49%)
n and % of Total	0 (0%)	3 (1.4%)	6 (2.9%)	76 (36.5%)	123 (59.1%)	208 (100%)
Positive Differences						56
Negative Differences						26
Ties						126
Wilcoxon-Signed Rank Test Z Statistic ^b c^						3.63***
*3. Most people do NOT feel comfortable talking about death/dying*						
Strongly Disagree	0 (0%)	0 (0%)	0 (0%)	0 (0%)	1 (0.5%)	1 (0.5%)
Disagree	0 (0%)	3 (1.4%)	2 (1%)	1 (0.5%)	0 (0%)	6 (2.9%)
Not Sure	1 (0.5%)	1 (0.5%)	7 (3.4%)	10 (4.8%)	0 (0%)	19 (9.1%)
Agree	0 (0%)	10 (4.8%)	8 (3.8%)	106 (51%)	26 (12.5%)	150 (72.1%)
Strongly Agree	0 (0%)	1 (0.5%)	0 (0%)	11 (5.3%)	20 (9.6%)	32 (15.4%)
n and % of Total	1 (0.5%)	15 (7.2%)	17 (8.2%)	128 (61.5%)	47 (22.6%)	208 (100%)
Positive Differences						40
Negative Differences						32
Ties						136
Wilcoxon-Signed Rank Test Z Statistic ^b c^						−0.34*ns*
*4. Death/dying is presented as a normal part of life in the mainstream media*						
Strongly Disagree	9 (4.3%)	6 (2.9%)	1 (0.5%)	0 (0%)	0 (0%)	16 (7.7%)
Disagree	13 (6.3%)	85 (40.9%)	9 (4.3%)	11 (5.3%)	2 (1.0%)	120 (57.7%)
Not Sure	0 (0%)	21 (10.1%)	13 (6.3%)	5 (2.4%)	2 (1.0%)	41 (19.7%)
Agree	1 (0.5%)	4 (1.9%)	9 (4.3%)	10 (4.8%)	5 (2.4%)	29 (13.9%)
Strongly Agree	0 (0%)	1 (0.5%)	0 (0%)	1 (0.5%)	0 (0%)	2 (1.0%)
n and % of Total	23 (11.1%)	117 (56.3%)	32 (15.4%)	27 (13%)	9 (4.3%)	208 (100%)
Positive Differences						41
Negative Differences						50
Ties						117
Wilcoxon-Signed Rank Test Z Statistic^b c^						0.25 *ns*
*5. Social media provides different perspectives to mainstream media on death/dying*						
Strongly Disagree	0 (0%)	0 (0%)	0 (0%)	0 (0%)	0 (0%)	0 (0%)
Disagree	1 (0.6%)	3 (1.7%)	3 (1.7%)	8 (4.5%)	2 (1.1%)	17 (9.5%)
Not Sure	1 (0.6%)	4 (2.2%)	28 (15.6%)	43 (24.0%)	3 (1.7%)	79 (44.1%)
Agree	0 (0%)	2 (1.1%)	10 (5.6%)	55 (30.7%)	9 (5.0%)	76 (42.5%)
Strongly Agree	0 (0%)	1 (0.6%)	0 (0%)	2 (1.1%)	4 (2.2%)	7 (3.9%)
n and % of Total	2 (1.1%)	10 (5.6%)	41 (22.9%)	108 (60.3%)	18 (10.1%)	179 (100%)
Positive Differences						68
Negative Differences						21
Ties						90
Wilcoxon-Signed Rank Test Z Statistic^b c^						4.63***

^a^Each analysis was based on *n* = 208 participants who had complete data for the death attitude questions at both enrolment and at the conclusion of the MOOC, with the exception of the question about social media, which was based on 179 participants who answered this question at both time points

^b^The non-parametric Wilcoxon-Signed rank test was chosen to analyse repeated measures change over time due to the ordinal nature of the death attitude questions, and their skewed distributions. The highly skewed distributions violated the normal distribution data assumption for a parametric paired-samples t-test. Nonetheless, the conclusions from the paired-samples t-tests and Wilcoxon-Signed rank tests were the same

^c^*ns* not statistically significant; *** *p* < .001

Section 2 of Table 5 displays results for the attitude ‘I am comfortable talking about death/dying’. Again, a skew in attitudes towards the positive is evident at both enrolment and the end of the MOOC. Overall 87.9% agreed or strongly agreed that they feel comfortable talking about death at the start of the MOOC, and by the end of the MOOC 95.6% agreed or strongly agreed they felt comfortable. At enrolment there were 64 out of 1154 people who reported disagreeing or strongly disagreeing with the statement ‘I am comfortable talking about death/dying’. Of these people, 48 commenced the MOOC after enrolling, and 14 of them (29.2%) continued to actively participate in the MOOC to the end, where they completed the evaluation questions activity in the final week. Of the subsample of 14 who we have both pre- and post-MOOC death attitudes data for, it can be seen in Table 5 that 13 of the 14 (92.9%) became more comfortable talking about death at the conclusion of the MOOC (one person’s response remained the same). For example, of the 10 who reported at enrolment that they strongly disagreed to feeling comfortable talking about death, 7 of them (70%) reported that by the end of the MOOC, they strongly agreed that they now feel comfortable talking about death. Overall, the majority of responses stayed the same (*n* = 126), but more than twice as many positive changes occurred over time than negative changes (*n* = 56 versus *n* = 26). A Wilcoxon Signed Rank Test revealed a statistically significant increase in agreement with the statement that ‘I am comfortable talking about death/dying’ following participation in the Dying2Learn MOOC, *Z* = 3.63, *p* < .001, with a small effect size (*r* = .178). The mean score on the ‘I am comfortable talking about death/dying’ question increased from pre-MOOC (*M* = 4.25) to post-MOOC (*M* = 4.53), and the median score increased from pre-MOOC (*Md* = 4 ‘agree’) to post-MOOC (*Md* = 5 ‘strongly agree’).

Table 5 Section 3 displays results for the attitude ‘Most people do NOT feel comfortable talking about death/dying’. Of the 208 respondents, 78.4% either agreed or strongly agreed at both time points that most people do not feel comfortable talking about death. A clear pattern was not discernible on this attitude over time. A Wilcoxon Signed Rank Test revealed little change in agreement with the statement that ‘Most people do NOT feel comfortable talking about death/dying’ following participation in the Dying2Learn MOOC, *Z* = − 0.34, *p* > .05, with a negligible effect size (*r* = .02). The mean and median scores on this question did not change from pre-MOOC (*M* = 3.99; *Md* = 4) to post-MOOC (*M* = 3.99; *Md* = 4).

Table 5 Section 4 displays results for the question ‘Death/dying is presented as a normal part of life in the mainstream media’. Again, no clear pattern emerged on this attitude. A Wilcoxon Signed Rank Test revealed little change in agreement with the statement that ‘Death/dying is presented as a normal part of life in the mainstream media’ following participation in the Dying2Learn MOOC, *Z* = 0.25, *p* > .05, with a negligible effect size (*r* = .01). The mean and median scores on this question did not change from pre-MOOC (*M* = 2.43; *Md* = 2) to post-MOOC (*M* = 2.43; *Md* = 2).

Table 5 Section 5 displays results for the question ‘Social media provides different perspectives to mainstream media on death/dying’. Half (*n* = 90, 50.3%) of the responses to this question stayed the same before and after the MOOC (e.g. 30.7% agreed to this statement at both time-points). Nonetheless, three times as many positive changes occurred over time than negative changes (*n* = 68 versus *n* = 21; 38% compared to 11.7%). A Wilcoxon Signed Rank Test revealed a statistically significant increase in agreement with the statement that ‘Social media provides different perspectives to mainstream media on death/dying’ following participation in the *Dying2Learn* MOOC, *Z* = 4.63. *p* < .001, with a small-to-medium effect size (*r* = .245). The mean score on this question increased from pre-MOOC (*M* = 3.41) to post-MOOC (*M* = 3.72), and the median score increased from pre-MOOC (*Md* = 3 ‘not sure’) to post-MOOC (*Md* = 4 ‘agree’).

In post-hoc analyses we examined whether the change over time in agreement to the social media question varied depending on the age group of the participant. The file was split into ‘under 40’ and ‘40 and over’ age groups to allow comparison of these ‘internet age’ generations. For the under 40 age group, a Wilcoxon Signed Rank Test revealed little change in agreement with the statement that ‘Social media provides different perspectives to mainstream media on death/dying’ following participation in the Dying2Learn MOOC, *Z* = 0.96, *p* > .05, with a small effect size (*r* = .10). The mean and median scores on this question changed little from pre-MOOC (*M* = 3.45; *Md* = 4) to post-MOOC (M = 3.60; *Md* = 4). By contrast, the Wilcoxon Signed Rank Test for the age group aged 40 and over revealed a statistically significant increase in agreement with the statement that ‘Social media provides different perspectives to mainstream media on death/dying’ following participation in the *Dying2Learn* MOOC, *Z* = 4.94, *p* = .000, with a medium effect size (*r* = .299). The mean score on this question increased from pre-MOOC (*M* = 3.35) to post-MOOC (*M* = 3.76), and the median score increased from pre-MOOC (*Md* = 3 ‘not sure’) to post-MOOC (*Md* = 4 ‘agree’). These findings suggest that older MOOC participants were more likely than younger students to have their attitudes towards social media perspectives changed as a result of participating in the Dying2Learn MOOC.

Table 6 examines socio-demographic differences in participants’ responses to the death attitude questions at enrolment and at the conclusion of the MOOC. Overall, socio-demographic characteristics did not have a huge impact on responses to these questions. Responses to the death attitude questions did not differ depending on the health professional status of the participant at either pre- or post-MOOC. Those who self-identified as health professionals did not report significantly more positive attitudes towards death before or after the MOOC. There were also no significant differences on death attitudes based on gender. In contrast, holding a university qualification did impact on participants responses to certain death attitudes. At enrolment, university qualified participants were slightly more likely than those without a university degree to agree that death is a normal part of life (*M* = 4.63 vs *M* = 4.50, *Z* = 4.09, *p* = .000), and that most people don’t feel comfortable talking about death (*M* = 3.99 vs *M* = 3.90, *Z* = 2.73, *p* = .006). At enrolment, university qualified participants showed more disagreement than non-qualified participants with the statement that ‘death is presented as a normal part of life in mainstream media’ (*M* = 2.26 vs *M* = 2.68, *Z* = − 7.55, *p* = .000), and this was still the case at the end of the MOOC (*M* = 2.21 vs *M* = 2.88, *Z* = − 4.38, *p* = .000). At the end of the MOOC, participants who did not have a university qualification showed more agreement to the statement that ‘social media provides different perspectives to mainstream media on death’ than did university qualified participants (*M* = 3.97 vs *M* = 3.59, *Z* = − 3.25, *p* = .001).

**Table 6:** Attitudes Toward Death by Socio-Demographic Characteristics ^a^

	*Health Occupation* ^*b*^	*University Qualified* ^*b*^	*Gender* ^*b*^	*Age* ^*c*^	*SEIFA Disadvantage* ^*c*^
	*Z (n)*	*Z (n)*	*Z (n)*	*r (n)*	*r (n)*
*Pre-MOOC Death Attitudes*					
Death is a normal part of life	0.44 (1154)	4.09*** (1154)	−1.05 (1140)	.05 (1148)	.02 (1078)
I am comfortable talking about death/dying	−1.38 (1154)	1.23 (1154)	−0.97 (1140)	.14*** (1148)	.01 (1078)
Most people do NOT feel comfortable talking about death/dying	1.75 (1154)	2.73** (1154)	−0.28 (1140)	.03 (1148)	.03 (1078)
Death/dying is presented as a normal part of life in the mainstream media	−0.17 (1154)	−7.55*** (1154)	0.73 (1140)	−.05 (1148)	−.14*** (1078)
Social media provides different perspectives to mainstream media on death/dying	0.64 (928)	−0.73 (928)	1.13 (915)	−.03 (922)	−.04 (877)
*Post-MOOC Death Attitudes*					
Death is a normal part of life	0.45 (208)	−0.22 (208)	−0.38 (206)	.09 (205)	−.06 (200)
I am comfortable talking about death/dying	−0.75 (208)	−1.35 (208)	−1.75 (206)	.18** (205)	−.19**(200)
Most people do NOT feel comfortable talking about death/dying	−0.78 (208)	0.61 (208)	−0.05 (206)	.21** (205)	.14 (200)
Death/dying is presented as a normal part of life in the mainstream media	1.39 (208)	−4.38*** (208)	0.82 (206)	−.02 (205)	−.13 (200)
Social media provides different perspectives to mainstream media on death/dying	−0.53 (208)	−3.25*** (208)	0.72 (206)	.06 (205)	−.09 (200)

^a^Pre-MOOC death attitudes data was provided by *n* = 1154, with the exception of the social media question, which was only presented to *n* = 928. *N* = 208 participants had socio-demographic data at enrolment and death attitudes data at the conclusion of the MOOC. A small number of participants had missing data on gender and age. SEIFA Disadvantage Index is only available for *n* = 1078 participants who provided an Australian postcode for their location at enrolment

^b^The non-parametric Mann-Whitney U test was used to analyse group differences due to the ordinal nature of the death attitude questions, and their skewed distributions. Nonetheless, the conclusions from the Independent samples t-tests and non-parametric Mann-Whitney U tests were the same

^c^Spearman’s Rank-Order Correlation was used to analyse associations due to the ordinal nature of the death attitude questions. Conclusions using Pearson’s correlations were very similar

*Note.* ** *p* < .01; *** *p* < .001

In Table 6 it can also be seen that participant age had a small but statistically significant positive association with feeling comfortable talking about death at both the beginning (*r* = .14) and the end (*r* = .18) of the MOOC. Older participants showed greater agreement with reporting they feel comfortable talking about death. At the end of the MOOC, there was also a significant small positive association between age and agreement with the statement that most people don’t feel comfortable with the talking about death (*r* = .21), with older participants showing greater endorsement of this belief. Regarding a participant’s socio-economic status, the SEIFA disadvantage index for participants’ Australian location held small negative associations with two of the death attitudes. At enrolment, those living in more advantaged SEIFA areas were less inclined to agree that death is presented as a normal part of life in mainstream media (*r* = −.14). At the end of the MOOC, higher levels of SEIFA area advantage were associated with less agreement that they feel comfortable talking about death (*r* = −.19). All other associations were not significant.

## Discussion

This study showed that a new approach to engaging with the community about death and dying using a MOOC platform was both valuable and surprising. Our original expectation for enrolments was around 250 people based on activity in other university MOOCs, so the actual enrolment of 1156 was unexpected. The numbers showed that there is both an interest in and a willingness to discuss death and dying, and to discuss death and dying in an online environment. The MOOC activity indicators showed that many participants were active not only in viewing resources and completing set tasks, but also were involved in making comments and commenting on others posts. During the MOOC nearly 10,000 comments were made. People not only shared personal experiences but reflected on how the topics were shaping their views and perceptions:

*I think that we struggle to describe death because we struggle to accept that we are all going to die. Somehow it’s seen as a failure... “lost her battle..*etc.*” even “sorry for your loss” talks about a lack. What is lost is the physical relationship and we place a lot of sway on the physical. (001 4/7/16).*

*I think there is that irrational idea that if we ignore death it won’t happen or, more to the point, if we talk about and plan for it we tempt fate. (002 5/7/16*).

*I think the more comfortable you are in yourself to discuss it will hopefully reflect in discussing with others and make them feel more comfortable. (003 31/7/16).*

The *Dying2Learn* MOOC was small by MOOC standards where course numbers can range up to hundreds of thousands and 25,000 would be seen to be an average MOOC size [27]. However, there are challenges in managing large numbers of participants. Our general approach to MOOC learning had been agreed during course development and it positioned participants as active providers of knowledge and co-contributors to the learning conversation. The topic leaders intended to maintain a “watching brief” allowing participants to respond to the material that had been curated for each topic week and to explore the issues embedded in the content and activities. This is a common approach taken in constructivist MOOCs reflecting adult learning principles and the reality of large online cohorts [28]. However, acknowledging participation and contribution is an important component of creating a supportive learning environment so with over 1100 participants even just reading and liking comments was time consuming. Also, as with any first experience of a new technology, there were technical issues that needed to be resolved. These challenges ranged from sorting out problems with different internet browsers to helping inexperienced technology users with basic online functions. These were dynamic problems that had not been anticipated and needed to be dealt with during the conduct of the course.

While there is diversity in how MOOCs define completion and participation, [29] reports indicate that completion rates vary from 0.7 to 52.1%, with a median value of 12.6%. For a participant to gain a certificate indicating they had participated in the *Dying2Learn* MOOC, they needed to have completed at least 10% of the activities and posted at least one comment. MOOCs are not like other university courses and people register for MOOCs for many different reasons. Some people who register for MOOCs intend to complete all the assessment for the course while many are simply having a look around or doing the bits that interest them. As free and open courses, participants have greater freedom than in more formal education courses and as a result there may be less commitment to “completion”. Overall however, participation in the *Dying2Learn* activities was strong with 30% to 40% of students completing each of the activities in the four topic weeks.

We were also surprised given the promotional messaging and the choice of marketing channels that nearly 70% of participants were health professionals. It is however, worth noting that over 350 non-health professionals or members of the “general public” did enrol which was more than our initial expectation of the total enrolment. Data analysis did not show that there was a difference in death attitudes between health professionals or non-health professionals either pre- or post-MOOC. We were interested to note that during the course, most participants engaged as individuals rather than in a professional role. Often responses would highlight personal experiences and they demonstrated that they were keen to learn about death and dying outside of their professional lens. This could suggest that the health workforce has identified a need to be able to more openly discuss death and dying. This may reflect a growing awareness that “palliative care is everyone’s business” as the population ages and palliative care needs increase.

*… for me death is very real. Both professionally and personally. I am able to deal with my professional deaths, I can talk to other people close to me about personal deaths but I cannot face those deaths on my own. (004 16/7/16).*

*Living in a small community means that many community clients & aged care residents that I work with are known to me, making palliative care/talking about death very personal. (005 22/7/16).*

Participants commencing in the MOOC were predominantly a cohort who were comfortable talking about death and who saw death as a normal part of life. Participating in the MOOC retained these positive views and for those who were less comfortable at the beginning participating in the MOOC made them feel more comfortable talking about death and dying. However, while this cohort themselves felt comfortable in discussing death and dying, this was not something they felt that most people were comfortable doing. What is unclear is whether it is only perception that others are less comfortable talking about death and dying or whether there are indeed groups who are actually less comfortable discussing death and dying and what is the impact of this. For the small proportion (5.5%) in the MOOC who did not feel comfortable talking about death and dying, their perception of how others viewed death and dying differed to those who were comfortable. One in five of these participants disagreed that most people do not feel comfortable talking death and dying, that is, they felt others may be more comfortable talking about death and dying while they were not. For this group believing others were more comfortable might have been a prompt to participate in the MOOC hoping to feel more comfortable talking about death and dying.

Participation in the MOOC seemed to reinforce and strengthen the view that death is a normal part of life and the individual’s comfort in talking about death and dying. Following the course, participants tended to agree social media provides differences perspectives on death and dying and this was more strongly seen in older age groups. This may have resulted from exposure to a wider range of social media representations and discussions of death and dying. Given that older people are moving rapidly into social media this may provide a channel for messaging around the importance of conversations around death and dying.

In assessing the value of the MOOC, it is clear that the platform was able to provide an environment that enabled open and supportive discussion around death and dying. A considerable number of people were willing to enrol and to participate. As with most MOOCs, there was a predominance of participants with university qualifications which may in this case have also reflected the large proportion of health professionals. Nonetheless, age did not appear to be a barrier with people from 16 to 84 years enrolled. Similarly, the platform enabled engagement across the whole of Australia mitigating the impact of geography on participation. Participants also came from both advantaged and less advantaged socio economic backgrounds, and from urban, rural and remote areas. Given that death is experienced by all in society, knowledge of approaches that support inclusion across all of the community are particularly important. It is worth noting that those living in significantly lower socio-economic (SEIFA) areas were more likely to commence MOOC participation once enrolled, suggesting a particular value of these online learning platforms in reaching those living in less advantaged areas. Given evidence of an association between lower socio-economic status and lower health literacy [30–32], the potential of online courses such as the Dying2Learn MOOC to facilitate participation in health related activities that can build knowledge and understanding should be explored. The evaluation data indicates that the participants became more comfortable discussing death and dying, developed a greater understanding of death and gained personal insights into their own beliefs. The MOOC also provided a novel opportunity to gain access to different views and perceptions of death and dying. Further analyses of these comments and views will be used to inform the development of CareSearch resources to support health professionals and those with palliative care needs. It appears that MOOCs may have a valuable role to play in enabling participatory learning that is self-paced and interactive and allows learners to select the elements of interest to them. This would be of particular value for community members in terms of its open and free access and its ability to create informal learning options including videos. Given the interest and engagement shown by the participating health professionals, they may also have a role as part of the health education landscape.

There are some important limitations associated with this study that should be noted. All participants were self-selected. It is likely that many of the participants were already interested in issues around death and dying and willing to engage with the topic. The cohort was highly educated and not representative of the whole population. While there was a pre-post measure, there was no control group, so we cannot be sure of a causal relationship between participation in the MOOC and improvements in death attitudes. The death attitudes items were not a standardised scale. Only some of the participants completed the evaluation and death attitudes questions at the end of the MOOC and they may not be a true or full representation of the whole cohort. There was however formal data collection associated with the conduct of the MOOC which provided sufficient numbers to undertake statistical analysis. It was also possible to extract data from the MOOC platform to increase the datasets available for analysis.

## Conclusions

The Dying2Learn MOOC provided a rare opportunity to explore community views and attitudes around death and dying within a learning environment rather than a health context. Enrolment rates demonstrated significant community interest and willingness to participate. Those who enrolled in the course were generally active with large number of participants viewing pages and completing activities. The completion rate of activities remained strong at around 30–40% across the four modules. The group who chose to participate included a large cohort of health professionals and the whole cohort was comfortable talking about death and dying at commencement. Even so, and despite the ceiling effect, there was an increase in the comfort in talking about death and dying at the end of the course. In summary, the *Dying2Learn* MOOC provided an opportunity to capture community views and perceptions around death and dying which will inform the development of palliative care resources and information.

## Abbreviations

COPD: Chronic obstructive pulmonary disease
MOOC: Massive open online course
SEIFA: Socio-economic index for areas index of relative socio-economic disadvantage
